# MicroRNA-181a-5p prevents the progression of esophageal squamous cell carcinoma *in vivo* and *in vitro* via the MEK1-mediated ERK-MMP signaling pathway

**DOI:** 10.18632/aging.204028

**Published:** 2022-04-25

**Authors:** Mingbo Wang, Chao Huang, Wenda Gao, Yonggang Zhu, Fan Zhang, Zhenhua Li, Ziqiang Tian

**Affiliations:** 1 Department of Thoracic Surgery, The Fourth Hospital of Hebei Medical University, Shijiazhuang 050000, China

**Keywords:** ESCC, miR-181a-5p, MEK1

## Abstract

MicroRNAs (miRNAs) have been revealed to play a crucial role in oncogenesis of esophageal squamous cell carcinoma (ESCC). However, the biological role of miR-181a-5p in ESCC is currently less explored. The current study was designed to assess whether miR-181a-5p affects ESCC progression and further investigate relevant underlying mechanisms. Based on the data of GSE161533, GSE17351, GSE75241 and GSE67269 downloaded from GEO database, MAP2K1 (MEK1) was revealed to be one overlapping gene of the top 300 DGEs. Additionally, using the predicting software, miR-181a-5p was projected as the presumed target miRNA. Immunohistochemical staining and RT-qPCR research revealed that miR-181a-5p expression was decreased in human tumor tissues relative to surrounding peri-cancerous tissues. In an *in vivo* experiment, miR-181a-5p mimics could inhibit tumor growth and metastasis of ESCC. Gene expression profiles in combination with gene ontology (GO) and KEGG pathway analysis revealed that MAP2K1 (MEK1) gene and ERK-MMP pathway were implicated in ESCC progression. MiR-181a-5p mimics inhibited the activity of p-ERK1/2, MMP2 and MMP9 *in vivo*, as shown by Western blotting and immunohistochemistry labeling. There were no variations in the expression of p-P38 and p-JNK proteins. Additionally, miR-181a-5p mimics lowered p-ERK1/2, MMP2 and MMP9 levels in ECA109 cells, which were restored by MEK1-OE lentivirus. MEK1-OE Lentivirus significantly reversed the function induced by miR-181a-5p mimics in ECA109 cells. Moreover, further investigation indicated that the capability of migration, invasion and proliferation was repressed by miR-181a-5p mimics in ECA109 cells. In short, repressed ERK-MMP pathway mediated by miR-181a-5p can inhibit cell migration, invasion and proliferation by targeting MAP2K1 (MEK1) in ESCC.

## INTRODUCTION

Esophageal squamous cell carcinoma (ESCC) is one of the most common malignant tumors worldwide, particularly in North-Central China [1–3]. Despite recent improvements in the prevention and treatment with radical esophagectomy and systemic chemoradiotherapy, the overall survival rate of advanced ESCC patients remains low owing to the high ability of invasion and metastasis [4–6]. However, the molecular pathways causing ESCC invasion and metastasis remain largely unknown. Thus, there is an urgent clinical need to understand the molecular pathways behind increasing metastasis in order to develop novel therapeutic options for ESCC. Matrix metalloproteinases (MMPs) play a crucial role in the degradation of the extracellular matrix (ECM), which leads to cancer invasion and metastasis [7, 8]. Among them, MMP-2 and MMP-9 are the major proteinases contributing to extracellular matrix degradation in the process of tumor cell migration [9, 10]. There is a large body of evidence that extracellular signal-regulated kinase 1/2 (ERK1/2), a significant member of mitogen-activated protein kinase (MAPK) family, is involved in MMP-2/9 regulation [10, 11]. Additionally, RAF may activate MEK1 and MEK2, resulting in the activation of extracellular signal-regulated kinases ERK1 and ERK2 [12–14].

MicroRNAs (miRNAs) are a class of short (19–22 nucleotides) noncoding RNAs involved in post-transcriptional regulation of their target genes, and they play crucial roles in cell proliferation, differentiation and migration [15–17]. In recent years, numerous studies have suggested that several miRNAs exert regulatory effects on cell invasion and migration in ESCC. Cui et al. indicated that miR-34a can suppress tumor progression by targeting oncogenic PLCE1 in ESCC [18]. Meanwhile, miR-455-5p has been found to be a tumor-suppressor miRNA that can inhibit the proliferation and migration of ESCC ECA109 cells by targeting Rab31 [19]. Chen et al. indicated that inhibition of miR-21 can inhibit tumor growth in ESCC [20]. Likewise, miR-30b-5p has been observed to function as a tumor suppressor miRNA in ESCC suggesting its potential therapeutic value [21]. Nevertheless, the biological role of miR-181a-5p in ESCC is currently less explored. Therefore, the aim of the present study was to investigate the biological role of miR-181a-5p in ESCC *in vivo* and *in vitro*, and to elucidate whether the effect of miR-181a-5p on ESCC is mediated by the ERK/MMP2/9 pathway.

## RESULTS

### The implication of MAP2K1 and miR-181a-5p in ESCC

**Figure 1:**
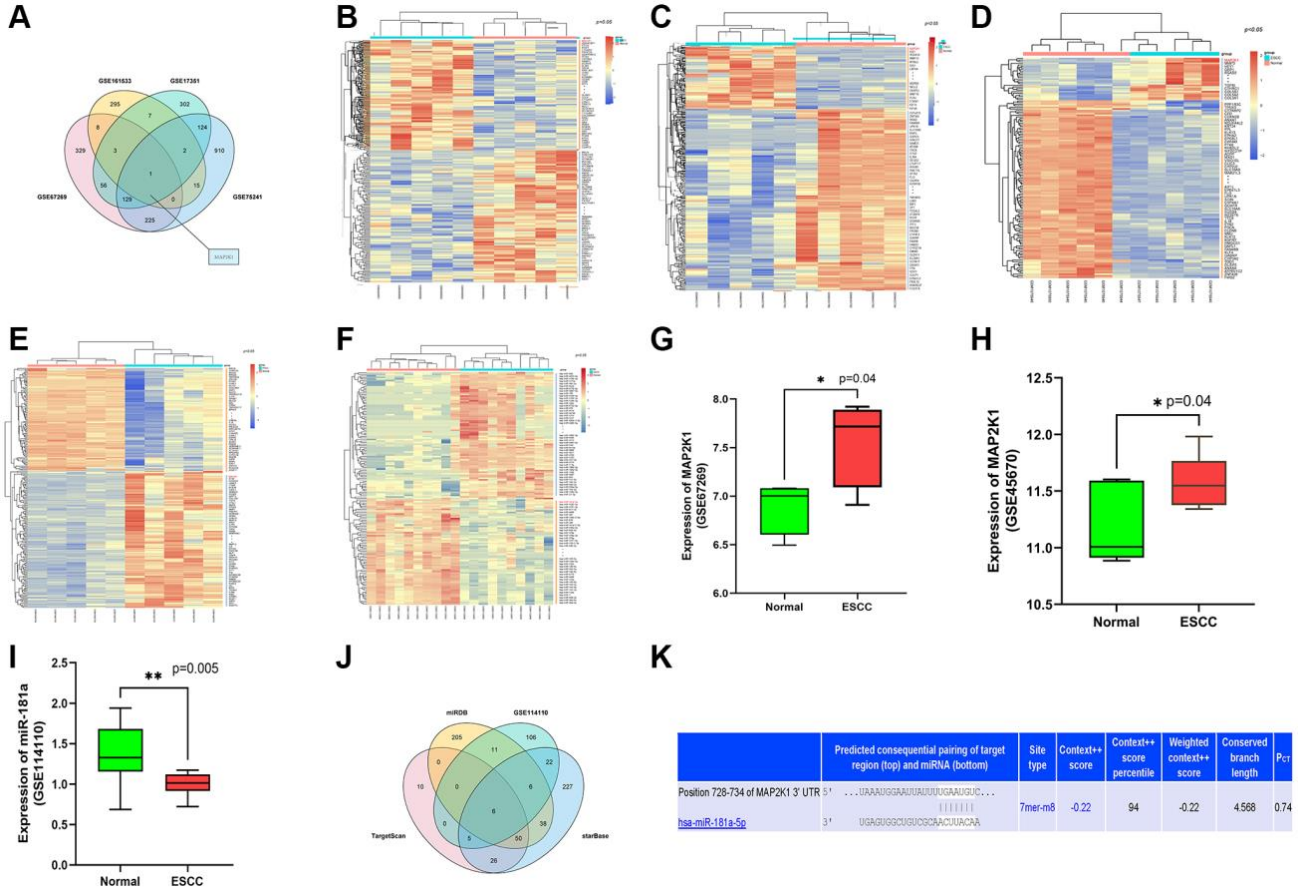
**MiR-181a-5p was predicted as an applicant miRNA that affected the development of ESCC by targeting MAP2K1 (MEK1).** (**A**) The top 300 DEGs determined from GSE161533, GSE17351, GSE75241 and GSE67269 datasets; (**B**–**E**) The heat map depicted the top 100 DEGs from GSE161533, GSE17351, GSE75241 and GSE67269 datasets; (**F**) The heat map depicted the top 100 DEMs from GSE114110 (**G**) the expression of MAP2K1 in ESCC in the GSE67269 dataset; (**H**) the expression of MAP2K1 in ESCC in the GSE45670 dataset; (**I**) the expression of miR-181a in ESCC in the GSE114110 dataset; (**J**) the top 100 miRNAs predicted from TargetScan, miRDB, and starBase. (**K**) miR-181a-5p was found had targeted binding regions with MAP2K1 (MEK1).

We analyzed gene expression profiles from Gene Expression Omnibus (GEO) database, GSE161533, GSE17351, GSE75241 and GSE67269, which were comprised of ESCC samples. DEGs were identified in these datasets with *P* < 0.05 and |logFC|>2. Following that, the top 300 DEGs in both datasets were determined. Among these DGEs, only one overlapped gene, MAP2K1 (MEK1), was discovered (Figure 1A). Heatmaps of the top 100 DEGs in GSE16153 (Figure 1B) GSE17351 (Figure 1C) and GSE67269 (Figure 1D). and GSE75241 (Figure 1E). Heatmap was depicted with the differentially expressed miRNA (DEMs) of GSE114110 (Figure 1F). MAP2K1 was identified as highly expressed gene in ESCC from the data in GSE67269 (Figure 1G) and GSE45670 (Figure 1H). The high expression of MAP2K1 in these datasets suggested that MAP2K1 might affect the development of ESCC. miR-181a was identified as low expressed microRNA in ESCC from the data in GSE114110 datasets (Figure 1I). The low expression of miR-181a in these datasets suggested that miR-181a might affect the development of ESCC. Assumed miRNAs targeting MAP2K1 (MEK1) gene were predicted using TargetScan, miRDB, and starBase. Six common predictive miRNAs were identified by analyzing the top 100 miRNAs from each prediction website: hsa-miR-30a-3p, hsa-miR-30b-5p, hsa-miR-145, hsa-miR-21-3p, hsa-miR-181a-5p, and hsa-miR-455-5p (Figure 1J). By comparing prediction findings, the predicted binding sites of mir-181a-5p and map2k1 were queried using targetscan online database was used to discovery that miR-181a-5p had targeted binding sites with MAP2K1 (MEK1) (Figure 1K). Emerging evidence indicates that miR-30a-3p, miR-30b-5p, miR-145, miR-21-3p and miR-455-5p are differentially expressed in ESCC, but the role of miR-181a-5p in ESCC has so far not been investigated.

### MiR-181a-5p was downregulated in human ESCC tissues

**Figure 2:**
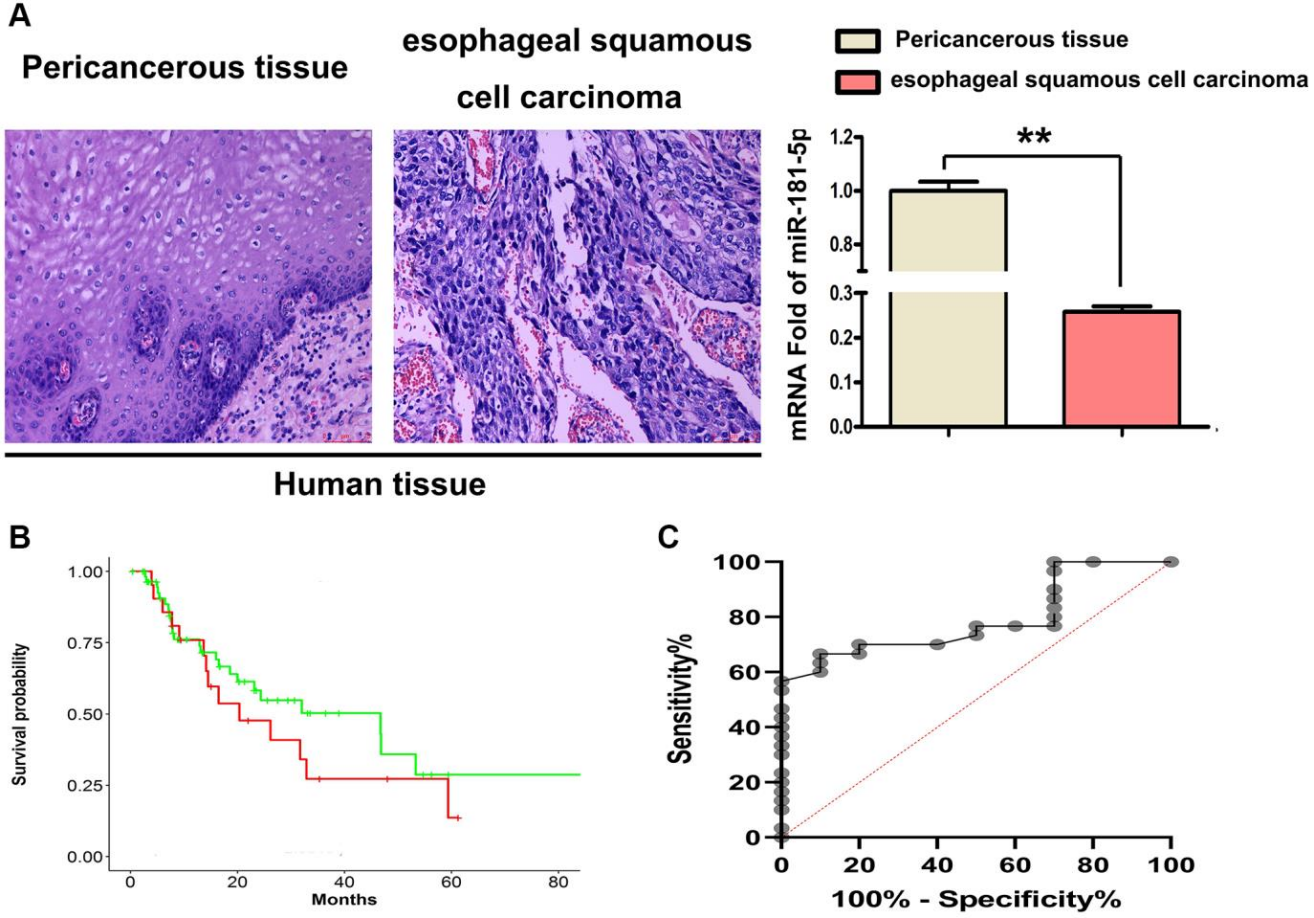
**MiR-181a-5p was downregulated in 4 paired human ESCC tissues.** (**A**) H&E staining was used to identified ESCC tissues and their corresponding adjacent peri-cancerous tissue specimens, RT-qPCR of miR-181a-5p expression in human ESCC tissues. ^*^P < 0.05, esophageal squamous cell carcinoma vs. peri-cancerous tissue. (**B**) Kaplan-Meier's method was used for survival analysis and miR-181 survival curve was drawn. (**C**) Kaplan-Meier analysis was used to depict the overall survival curves of patients with of ESCC from the data in GSE114110.

We used immunohistochemical labeling and RT-qPCR to examine the expression of miR-181a-5p in human ESCC tissues and their equivalent surrounding peri-cancerous tissues to determine the influence of ESCC on miR-181a-5p. As shown in Figure 2A, it was found that miR-181a-5p staining was positive in peri-cancerous sections, whereas small amount stained positive for miR-181a-5p in ESCC tumor sections. The relative expression level of miR-181a-5p was notably lower in the ESCC tumor specimens than in the adjacent peri-cancerous specimens (Figure 2A, *P* < 0.05). Kaplan-Meier’s method was used for survival analysis and miR-181 survival curve and shown in Figure 2B. The diagnostic value of miR-181a-5p in ESCC was also estimated using the receiver operating characteristic (ROC) curve. From the data in GSE114110, Kaplan-Meier analysis revealed that miR-181a-5p was related with greater overall survival (Figure 2C), suggesting that miR-181a-5p may play an important role in ESCC development.

### Upregulation of miR-181a-5p repressed the tumor growth and metastasis of ESCC *in vivo*

**Figure 3:**
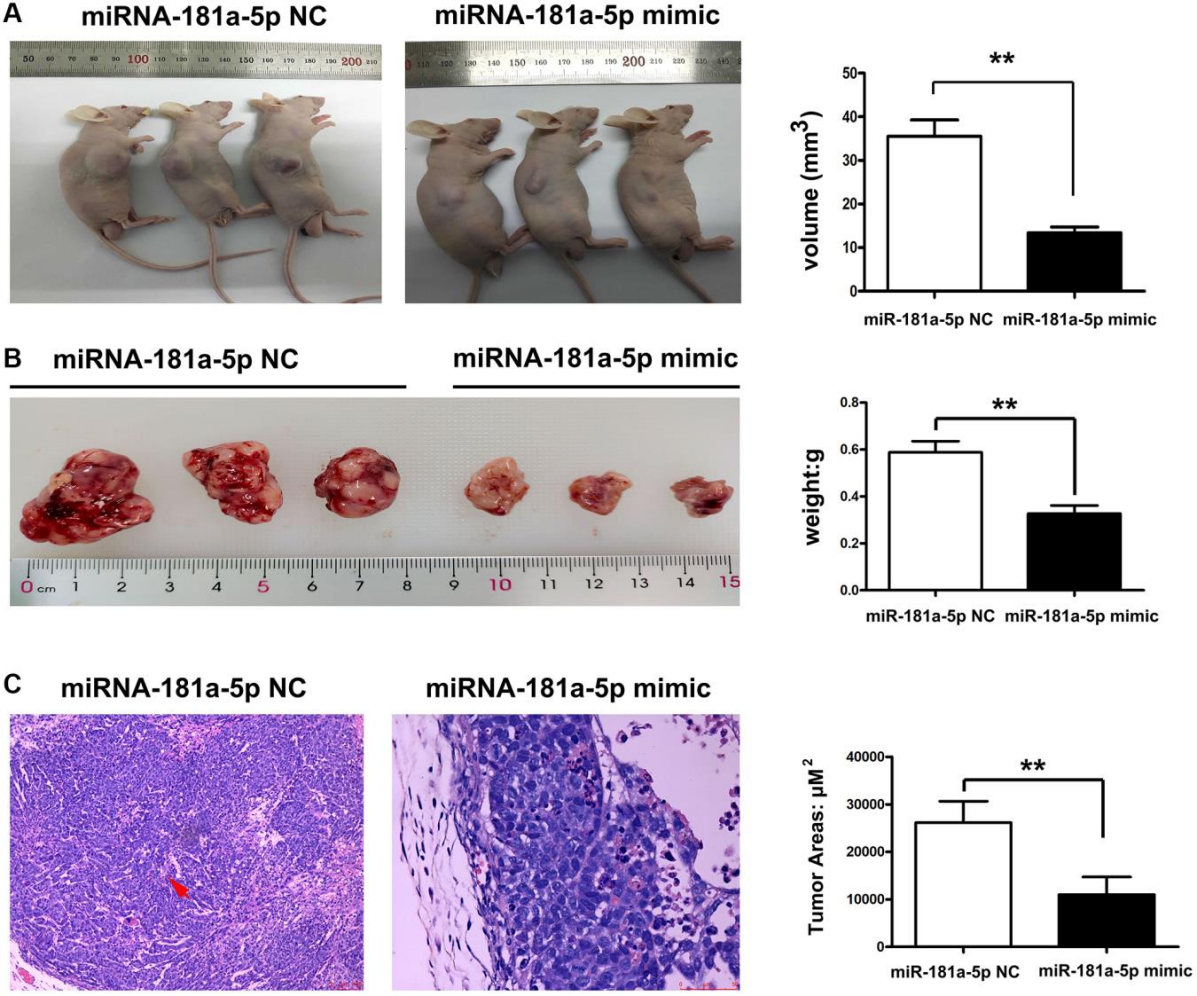
**Upregulation of miR-181a-5p inhibited tumor growth and metastasis of ESCC.** Nude mice were injected subcutaneously with ESCC cells and intratumorally injected with miR-181a-5p via. (**A**) The tumor volume in miR-181a-5p mimics and negative control mice; (**B**) the tumor weight in miR-181a-5p mimics and negative control mice; ^*^P < 0.05 miR-181a-5p mimics vs. the NC groups; (**C**) H&E staining in experimental mice; ^*^P < 0.05, ^**^P < 0.01 miR-181a-5p mimics vs. the NC groups.

To validate the effects of miR-181a-5p *in vivo*, tumor-bearing nude mouse models were established in this study. ECA109 cells were injected subcutaneously into the BALB/c nude mice. Tumors appeared seven days after injection. All of the animals were separated into two groups: miR-181a-5p mimics-control and miR-181a-5p mimics, with the latter receiving miR-181a-5p by intratumorally injection. Tumor growth was slower in the miR-181a-5p mimics group than in the miR-181a-5p mimics-control group (Figure 3A). Compared with the negative control group, the average tumor weight in the miR-181a-5p mimics group was significantly decreased (Figure 3A, *P* < 0.05). H&E staining was used to assess the histology of experimental mice. As indicated in Figure 3B and 3C, the negative control group had more dilated lymphatic arteries and tumor metastasis, but the size and area of the tumor were dramatically reduced in miR-181a-5p mimic group. The relative areas of tumor were further determined. The miR-181a-5p mimics significantly decreased the tumor areas compared with negative control (Figure 3B, *P* < 0.05). All the above data exhibited that the upregulation of miR-181a-5p repressed the tumor growth and metastasis of ESCC *in vivo*.

### The MAP2K1 (MEK1) gene and ERK-MMP pathway were implicated in ESCC progression

**Figure 4:**
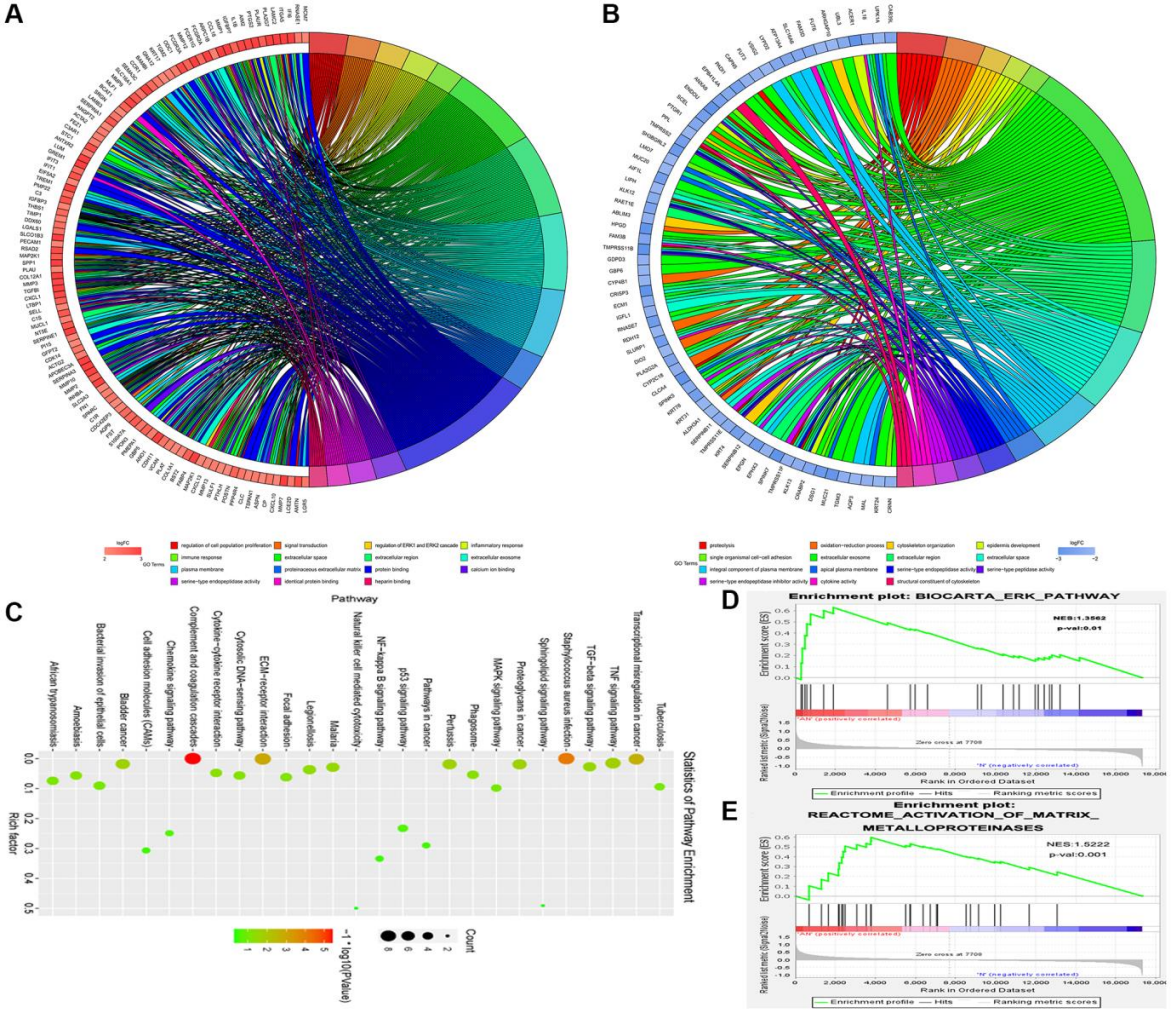
**The MAP2K1 (MEK1) gene and ERK-MMP pathway were implicated in ESCC progression.** Gene expression profiles in combination with gene ontology (GO) and KEGG pathway analysis was performed; (**A**) The results of the Go pathways from the data in GSE161533 dataset; (**B**) the results of the Go pathways from the data in GSE17351; (**C**) partial results of the KEGG pathways from the data in GSE17351; (**D** and **E**) the activation of matrix metalloproteinases signaling pathway and ERK signaling pathway were enriched in ESCC and appeared to be strongly correlated to with MEK1.

Gene expression profiles were combined with gene ontology (GO) and KEGG pathway analyses to further investigate the general impact of the MAP2K1 (MEK1) gene involved in ESCC. The string was used to find gene-enriched biological process items using GO pathway analysis. Results exposed that regulation of cell population proliferation, regulation of ERK1 and ERK2 cascade, and signal transduction were enriched pathways in ESCC from the data in GSE161533 dataset (Figure 4A). Figure 4B shows the results of the Go pathways from the GSE17351 data, which included single organismal cell-cell adhesion, proteolysis, and extracellular exosome. Figure 4C shows the incomplete results of the KEGG pathways derived from the GSE17351 data, including the MAPK signaling pathway. To further clarify the role of MAP2K1 (MEK1) in ESCC, GSEA was carried out to examine the two microarray datasets (GSE17351 and GSE161533) as biological relevance. As shown in Figure 4D and 4E, the results exposed that activation of matrix metalloproteinases signaling pathway and ERK signaling pathway were enriched in ESCC and appeared to be strongly correlated to with MEK1. These results implied that MAP2K1 (MEK1) activation was significantly associated with ERK-MMP signaling pathway in ESCC. This conclusion hypothesizes that the effects of miR-181a-5p expression and its target gene MAP2K1 (MEK1) on ESCC may be achieved through the ERK-MMP signaling pathway.

### Elevation of miR-181a-5p downregulated ERK1/2 to block the MMP2/9 signaling pathway *in vivo*

**Figure 5:**
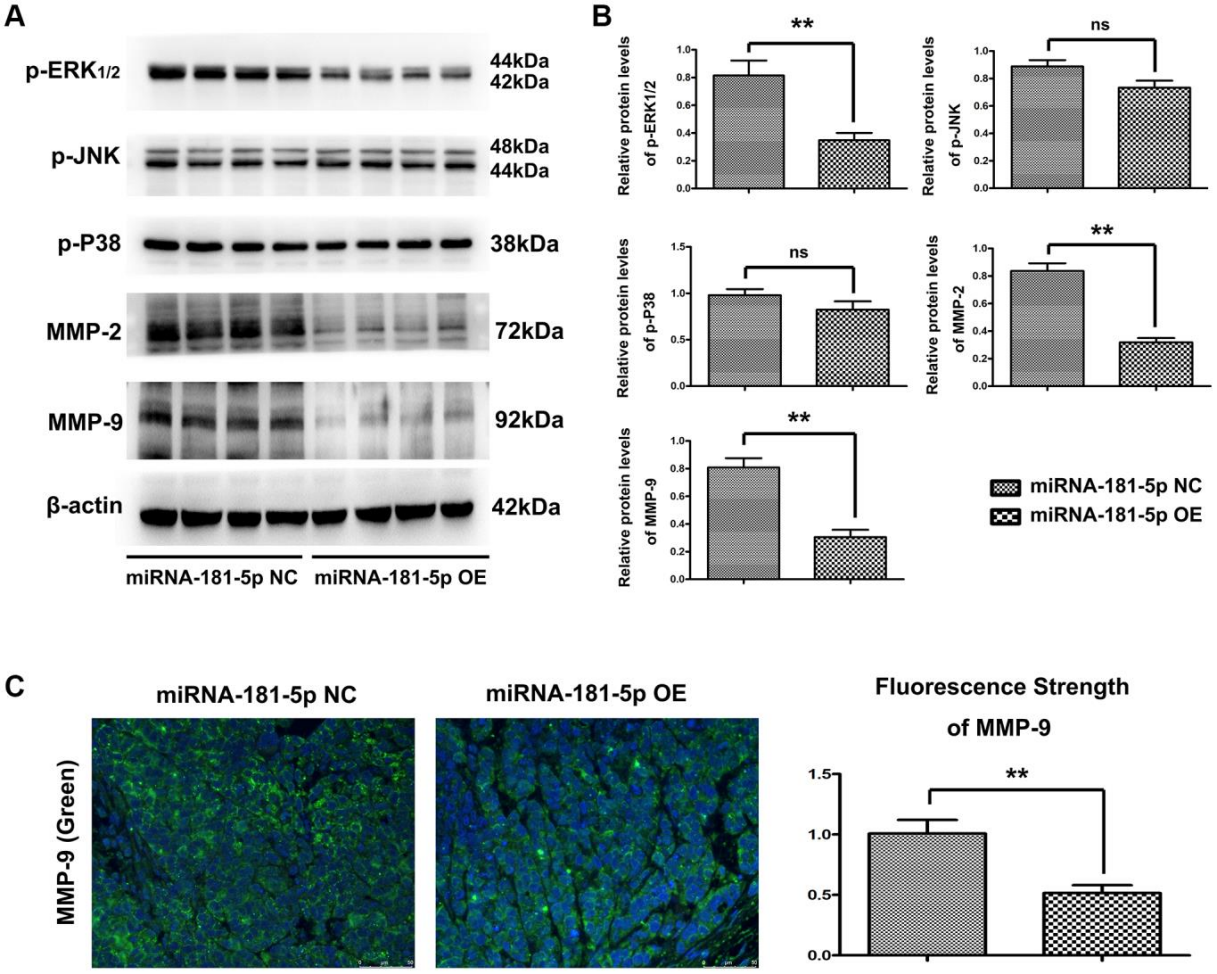
**Elevation of miR-181a-5p reduced p-ERK, MMP2 and MMP9 *in vivo*.** Nude mice were injected subcutaneously with ESCC cells and intratumorally injected with miR-181a-5p; (**A** and **B**) the protein expression of p-ERK1/2, p-JNK, p-P38, MMP2 and MMP9 as evaluated by Western blotting and RT-qPCR. ^*^P < 0.05 miR-181a-5p mimics vs. NC group; (**C**) Immunohistochemical staining of MMP9 in miR-181a-5p mimics group and in negative control group; the positive expression rate of MMP9 in miR-181a-5p mimics group and negative control group. ^*^P < 0.05, ^**^P < 0.01 miR-181a-5p mimics vs. NC group.

As the bioinformatics analysis suggested that MAP2K1 (MEK1) regulated the ERK-MMP pathway and miR-181a-5p played a crucial role in ESCC pathogenesis, we next performed Western blotting and quantitative analysis to the key genes of ERK-MMP pathway. We also looked into whether miR-181a-5p has a therapeutic effect on ERK1/2 and MMP2/9 in ESCC pathogenesis. Western blotting revealed that miR-181a-5p mimics decreased the protein expression of p-ERK1/2, MMP2, and MMP9 *in vivo* (Figure 5A). There were no variations in p-P38 or p-JNK protein expression, implying that increase of miR-181a-5p-induced MMP2/9 is mediated by ERK1/2 activation rather than P38 or JNK activation. Furthermore, the proteins for expression of p-ERK1/2, p-JNK, p-P38, MMP2 and MMP9 were further used to validate Western blotting results. As shown in Figure 5B, miR-181a-5p mimics significantly decreased the relative protein expression levels of p-ERK, MMP2 and MMP9 compared with miR-181a-5p negative control (*P* < 0.05). Furthermore, we verified the MMP9 activation by immunohistochemical staining. MiR-181a-5p mimics dramatically reduced MMP9 positive in peri-cancerous sections, according to the findings (Figure 5C), consistent with the Western blotting results. Collectively, these results indicated that miR-181a-5p elevation suppresses the activation of the ERK1/2-induced MMP2/9 pathway in ESCC, and the original data of western blot was shown in Supplementary Figure 1.

### MiR-181a-5p regulates ERK1/2, MMP2 and MMP9 expression *in vitro*

**Figure 6:**
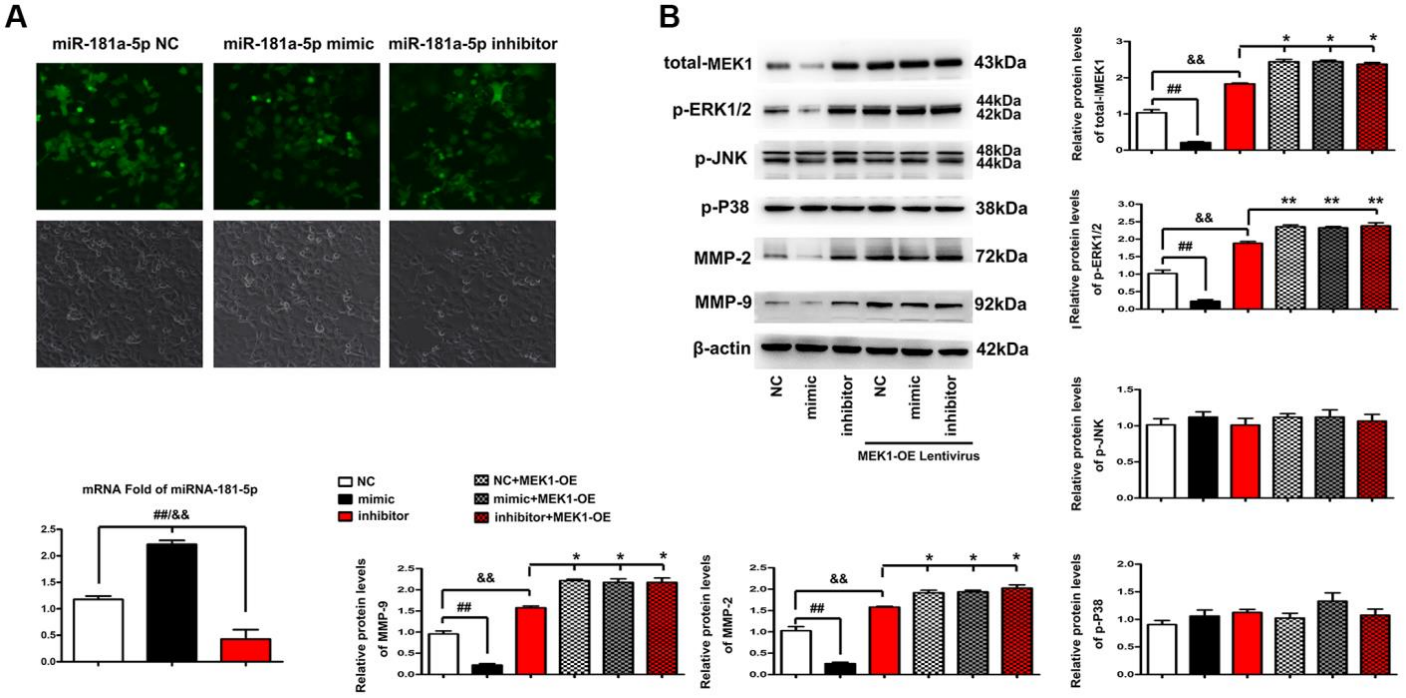
**Elevation of miR-181a-5p regulated ERK-MMP pathway in ESCC through MEK1.** (**A**) FISH was performed to identify the distribution and expression levels of miR-181a-5p in miR-181a-5p NC, miR-181a-5p mimics or inhibitor transfected ECA109 cells. The positive expression shows green fluorescence. The red arrows represent a tiny amount of fluorescence of miR-181a-5p. (**B**) Western blotting analysis of p-ERK1/2, p-JNK, p-P38, t-ERK1, MMP2 and MMP9 in miR-181a-5p NC, miR-181a-5p mimics or inhibitor transfected ECA109 cells. The p-ERK1/2, p-JNK, p-P38, t-ERK1, MMP2 and MMP9 protein levels were measured after co-transfection with miR-181a-5p mimics, miR-181a-5p NC or miR-181a-5p inhibitor and MEK1-OE Lentivirus in ECA109 cells. Different data were presented as mean ± S.D. (all *P* < 0.05).

To confirm that MEK1 is the downstream target of miR-181a-5p, miR-181a-5p mimics, NC or inhibitor were transfected into the ECA109 cells. The distribution and expression levels of miR-181a-5p in ECA109 cells were examined by FISH. MiR-181a-5p was found in most parts of the cytoplasm of ECA109 cells in the miR-181a-5p mimics group, whereas very little fluorescence was seen in the miR-181a-5p inhibitor group, as shown in Figure 6A. According to the results of RT-qPCR analysis, it was found that compared with the NC and miR-181a-5p inhibitor group, the mRNA expression of miR-181a-5p in ECA109 cells was significantly up-regulated in the miR-181a-5p mimics group (all *P* < 0.05). Subsequent analysis was performed to explain whether miR-181a-5p could affect the key genes in ERK-MMP pathway. The protein levels of p-ERK1/2, MMP2 and MMP9 in ECA109 cells were significantly decreased after transfection with miR-181a-5p mimics compared with NC and miR-181a-5p inhibitor (Figure 6B). Likewise, the phosphorylation levels of P38 and JNK, and the total level of ERK1 protein still remained unchanged (data not shown). Then, ECA109 cells were co-transfected with miR-181a-5p mimics, miR-181a-5p inhibitor or NC and MEK1-OE Lentivirus. Interestingly, it was found the expression levels of p-ERK1/2, MMP2 and MMP9 were recovered in miR-181a-5p mimics group. ERK1-OE Lentivirus reversed the decreased p-ERK1/2, MMP2 and MMP9 in ECA109 cells, indicating that the miR-181a-5p elevation suppressed the activation of the ERK1/2-induced MMP2/9 pathway in ESCC through MEK1 gene. According to the results of RT-qPCR (Figure 6B), it was found that compared with the miR-181a-5p inhibitor and NC group, the protein expression of p-ERK1/2, MMP2 and MMP9 in miR-181a-5p mimics group was significantly down regulated (all *P* < 0.05). Furthermore, ERK1-OE Lentivirus treatment reversed the decreased protein expression of p-ERK1/2, MMP2 and MMP9 in miR-181a-5p mimics group (all *P* < 0.05). Simultaneously, the phosphorylation levels of P38 and JNK, and the total level of ERK1 protein still remained unchanged. Altogether, miR-181a-5p elevation suppressed the activation of the ERK1/2-induced MMP2/9 pathway in ESCC hroughMEK1 gene, consistent with our bioinformatics analysis results, and the original data of western blot was shown in Supplementary Figure 2.

### Elevation of miR-181a-5p represses ESCC cell migration, invasion and proliferation

**Figure 7:**
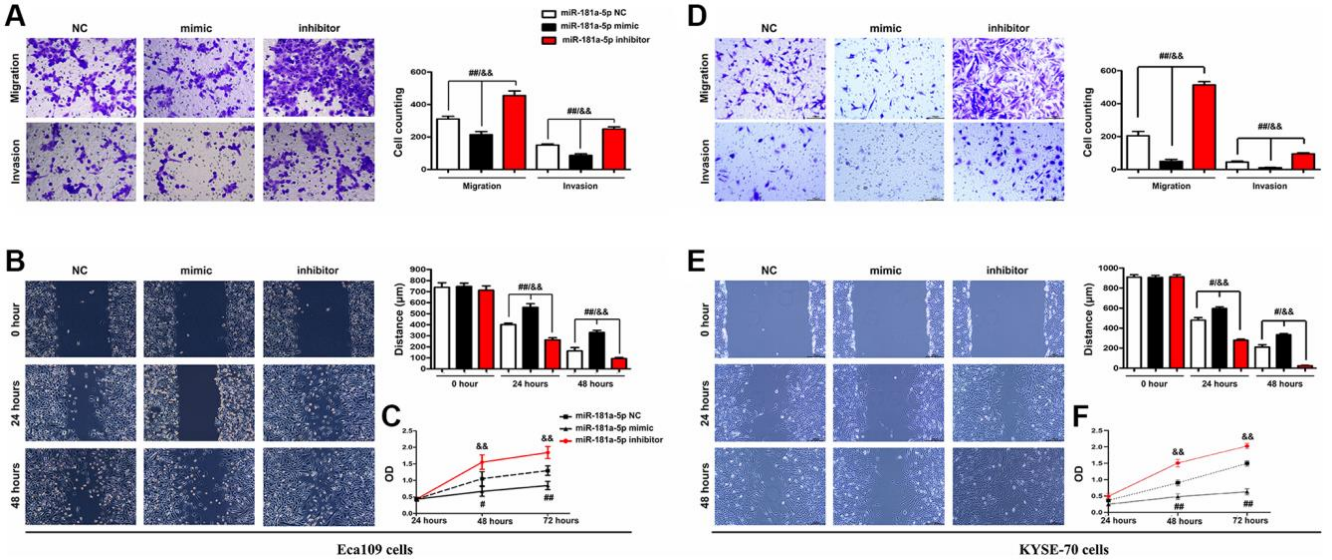
**Elevation of miR-181a-5p repressed ESCC cell migration, invasion and proliferation.** ECA109 and KYSE-70 cells were transfected with miR-181a-5p mimic, inhibitor or negative control (NC) for 48 hours; (**A**, **D**) Transwell invasion assay exposed that miR-181a-5p mimic inhibited cell migration and invasion in Eca109 (**A**) and KYSE-70 cells (**B**) Wound healing assay exposed that miR-181a-5p mimic inhibited cell migration. Representative images were obtained at 0, 24 and 48 h. RT-qPCR analysis of wound area in Eca109 (**C**) and KYSE-70 cells (**D**). The proliferation of ECA109 cells treated with miR-181a-5p mimic, inhibitor or negative control (NC) was measured by CCK8 assay in Eca109 (**E**) and KYSE-70 cells (**F**). ^*^P < 0.05, ^**^P < 0.01 miR-181a-5p mimics vs. NC group.

Transwell assays were performed to investigate the effect of miR-181a-5p on the migration and invasion of ESCC cells. The findings revealed that inhibiting miR-181a-5p induced an increase in cell migration and invasion ability in ECA109 cells. In contrast to the NC and the miR-181a-5p inhibitor, miR-181a-5p mimics greatly reduced cell migration and invasion in ECA 109 cells (Figure 7A) and KYSE-70 cells (Figure 7B). These results showed that elevation of miR-181a-5p mimics repressed ESCC cells migration and invasion, which were corroborated by the wound healing migration assay. Based on the wound healing assay, the altering in width of miR-181a-5p mimics was less apparent than that of the NC and miR-181a-5p inhibitor group at 24 and 48 hours after wounding. The ESCC cells in the miR-181a-5p inhibitor group, on the other hand, showed faster wound healing rates. RT-qPCR study of the wound region corroborated the findings. According to the findings, the relative wound area in the miR-181a-5p mimics was significantly reduced at 24 and 48 hours after wounding (all *P* < 0.05) compared to the miR-181a-5p inhibitor and NC group, the results of ECA 109 cells were shown in Figure 7C and KYSE-70 cells were shown in Figure 7D. The viability of ECA109 cells was significantly decreased in miR-181a-5p mimics group, while inhibition of miR-181a-5p significantly increased the proliferation of ESCC cells tested by CCK-8, and the results of ECA 109 cells were shown in Figure 7E and KYSE-70 cells were shown in Figure 7F, suggesting elevation of miR-181a-5p mimics inhibited the proliferation of ESCC cells. Collectively, these results suggested that elevation of miR-181a-5p represses migration, invasion and proliferation of ESCC cells.

## DISCUSSION

This study identified the effects of mir-181a-5p in the progression of esophageal cancer by *in vivo* and *in vitro* experiments, and we confirmed that the up-regulation of mir-181a-5p can reduce the expression of MEK1 and inhibit the transmission of ERK signal pathway, so as to inhibit the proliferation, migration and invasion of esophageal cancer cells.

Esophageal cancer is the main cancer in the world, and its occurrence and development is a complex process, involving the changes of many oncogenes and tumor suppressor genes [22]. A further understanding of the progression and metastasis mechanism of esophageal cancer can provide important research basis and theoretical basis for its diagnosis, treatment and prognosis. Although in recent years, it has been found that miRNA induces gene expression by binding to 5′UTR in the promoter region of the target gene, or inhibits gene expression by binding to 3′urt of the target gene, so as to degrade mRNA or prevent mRNA translation [23], interestingly, the expression level of mir-181a-5p is up-regulated or down-regulated in different cancers, This depends on the type and degree of differentiation of the tumor. In several studies on gastric cancer, liver cancer and breast cancer, we found that the expression of miR-181a-5p was up-regulated and promoted the progression of these cancers through different mechanisms [24–26]. In leukemia, oral squamous cell carcinoma and glioma, miR-181a-5p is considered to be a tumor suppressor, and its expression is down regulated by [27, 28]. These controversial observations suggest that the complexity of miRNA function varies significantly depending on the type of tumor. In our study, mir-181a-5p is down regulated in esophageal squamous cell carcinoma, whether in biological information mining, clinical specimen examination and animal experiments. It prevents its expression by binding with the target gene MAP2K1 (MEK1), thus affecting the proliferation and metastasis of esophageal cancer cells.

The signal cascade of JK-125 is the key to promote cell proliferation and metastasis, including the signal cascade of jk-125, which can promote the proliferation of tumor cells, MEK1 can also be used as a key signal node to regulate the expression of ERK1/2 and transmit signals to the nucleus [29]. The phosphorylation of MEK1 can activate ERK1/2 and promote the expression of transcription factors, jointly regulating a variety of cellular and biological functions in the development of cancer [30]. In our study, mir-181a-5p mimic can effectively reduce MEK1 and ERK1/2, while transfection of MEK1 overexpression lentivirus can reverse this effect. However, the expression of p-JNK and p-p38 are not affected by these treatments, indicating that mir-181a-5p plays a role through ERK signal pathway in esophageal cancer, rather than JNK signal pathway and p38MAPK signal pathway. As the main family members of MMPs, MMP2 and MMP9 play an important role in the invasion and metastasis of cancer cells. MMP2 can degrade the basement membrane, and MMP9 can induce tumor cells to infiltrate and migrate to surrounding tissues along the damaged basement membrane [31]. In this study, mir-181a-5p mimic can significantly reduce the expression, scratch and migration of MMP2/9 Invasion experiments confirmed that mir-181a-5p mimic could inhibit the motility of esophageal cancer cells, and the cells were transfected with MEK1 overexpression lentivirus at the same time, which was reversed, which further proved the inhibitory effect of mir-181a-5p on the motility of esophageal cancer.

In conclusion, this study found that the expression of mir-181a-5p was down regulated in esophageal cancer, effectively reduced the expression of MEK1, and mediated ERK signaling pathway to inhibit the proliferation, invasion and metastasis of esophageal cancer, so as to provide theoretical basis and research basis for the role of mir-181a-5p in the occurrence and development of esophageal cancer and the discovery of new treatment strategies.

## METHODS

### Bioinformatics

GSE161533, GSE17351, GSE75241 and GSE67269 datasets were downloaded from the GEO and. The differential analysis was selected with |logFC|>2 and *p* < 0.05 to obtain the differentially expressed genes. Cluster analysis was conducted by Cluster and TreeView software (USA). Survival analysis was conducted in by the “survival” package. GSEA was used for pathway enrichment analysis to study the association of target genes. Fisher’s exact test was used to screen the significant pathways. Statistical significance was accepted at *P* < 0.05.

### Human samples

We collected 7 fresh ESCC samples and 7 paired adjacent normal tissues (>5 cm away from the edge region of tumor) from 4 patients with ESCC at The Fourth Affiliated Hospital of Hebei Medical University. All specimens were embedded in paraffin wax and stored at −80°C until use. The human samples were obtained with informed consent and according to a protocol approved by the Ethics Committee of The Fourth Affiliated Hospital of Hebei Medical University, and written informed consent was obtained from each participant of this study.

### *In vivo* studies

Sixteen female BALB/c nude mice (aged 4–6 weeks) were purchased from Experimental Animal Center of Skbex Biotechnology (Henan, China) and fed with aseptic food and water. All animal experiments were approved by the Ethics Committee of Human and Animal Experiments of The Fourth Affiliated Hospital of Hebei Medical University, Shijiazhuang, Hebei, China. To establish the ESCC xenografts, 1 × 10^7^ Eca109 cells were injected subcutaneously to the right flank of each BALB/c nude mouse. When suffering obvious tumors, the mice were daily injected intratumorally with 100 nM miR-181a-5p mimics (*n* = 6) and corresponding negative controls (*n* = 6) every 2 days for 20 days. At the end of experiment, the mice were sacrificed, and the tumors tissues were resected for further experiments.

### Western blotting assay

Western blotting was performed to explore the protein expression levels of MEK1, ERK1/2, p-JNK, p-P38, p-ERK1/2, MMP2 and MMP9 as previously described [32]. The tissues and cells were lysed with RIPA lysis buffer (Beyotime) and centrifuged at 12000 rpm for 20 min at 4°C. The protein was loaded and separated by 10% SDS-PAGE and then transferred onto a polyvinylidene fluoride (PVDF) membrane (Roche Applied Science). After blocking with 5% skimmed milk at room temperature for 1 h, the membrane was incubated with the following primary antibodies at 4°C overnight. All antibodies were purchased from Abcam (USA). Then, the membrane was incubated with horseradish peroxidase-conjugated secondary antibodies for 1 h at room temperature. The protein bands were observed using multifunctional gel imaging system (6000 pro II, Guangzhou Biolight Biotechnology Co., Ltd., China).

### Immunofluorescence staining

Paraffin-embedded tissue sections (4 μm) were deparaffinized, rehydrated and stained as previously described [33]. The green fluorescent labeled secondary antibody was used to visualize protein expression after staining with anti-MMP9 (1:100). The intensity of immunohistochemistry staining was analyzed by two pathologists.

### Q-PCR

Total RNA was extracted from human tumor tissues and cells by TRIzol reagent (Ambion, USA) and reversely transcribed using a TaqMan MicroRNA Reverse transcription kit (Applied Biosystems, USA). The expression levels of miRNA-181-5p and mRNA were assessed with TaqMan miRNA analysis. U6 was taken as the internal reference for miRNA and mRNA, glyceraldehyde-3-phosphate dehydrogenase (GAPDH) was used as the internal reference. All the primer sequences were designed and synthesized by Bio Just Biomart Company (Wuhan, Hubei, China).

### Cancer cell lines and culture conditions

Human ECA109 cell lins and KYSE-70 cell line, cat No: AC340532, were obtained from the Tumor Cell Bank of Chinese Academy of Sciences or Shang Hai Ze Ye Biotechnology Co., Ltd (Shanghai, China). ECA109 cell lines were maintained in RPMI 1640 medium (Life Sciences, China) supplemented with 10% fetal bovine serum (FBS), 100 U/mL penicillin, and 1% streptomycin (Life Technologies, China) in a humidified incubator with 5% CO_2_. Lipofectamine 3000 was used to transfect NC, mir-181-5p mic and mir-181-5p inhibitor. Eca109 cells were digested and planted in six well plates with a density of about 50–60%. 50 nm NC, mir-181-5p mimic and mir-181-5p inhibitor were dissolved in 250 nm solution respectively μL in opti MEM serum-free medium, another 12 μL Lipofectamine 3000 (Invitrogen, l3000001) dissolved in 250 μL in opti MEM serum-free medium, place at room temperature for 5 minutes, then mix the two solutions, place at room temperature for 20 minutes, and add 1480 to the six well plate with pre laid cells μL of serum-free medium, then add the above mixture into the corresponding well, change to serum-containing medium after 4–6 hours, and continue to culture for 48 h–96 h.

NC: 5′-UUUGUACUACACAAAAGUACUG-3′; miR-181-5p mimic: 5′-AACAUUCAACGCUGUCGGUGAGU-3′; miR-181-5p inhibitor: 5′-ACUCACCGACAGCGUUGAAUGUU-3′.

### Transwell assay

A total of 5 × 10^3^ ECA109 and KYSE-70 cells were seeded in an upper chamber with serum-free medium. Concurrently, the lower chamber was filled with complete DMEM (Lonza, BE12-604F) containing 20% fetal bovine serum (FBS). After incubation for 24 h, the upper chamber was rinsed, fixed with 95% ethanol and stained with 0.1% crystal violet solution (in 20% methanol; 20 μL per well). The cells that migrated to the bottom surface of the upper chamber were depicted under an inverted optical microscope (Leica).

### Wound healing assay

The stably transfected ECA109 and KYSE-70 cells were seeded in 6-well culture plates. Upon reaching an 85–95% density, the cell monolayer was scratched with a pipette tip. After washing with culture medium to remove any suspended cells, ECA109 cells were cultured in fresh medium containing 1% FBS. The wound healing was observed at 0, 24 h and 48 h after scratching, and the wound area was calculated using ImageJ 1.44 (National Institutes of Health, USA).

### Cell count kit-8 (CCK-8)

The transfected ECA109 and KYSE-70 cells were seeded in 96-well culture plates at a density of 2 × 10^4^ cells/mL overnight and cultured at 37°C. Cells transfected with miR-181a-5p mimic, inhibitor or negative control (NC) were treated with 10 mg/mL cisplatin. At 0, 24 and 48 h of culture, 10 μL CCK-8 reagent (Beyotime Biotechnology) was added to each well for incubation at 37°C for 2 h. Cell survival was detected using the CCK-8 kit according to the manufacturer’s protocol.

### Statistical analysis

Statistical analysis was carried out using SPSS 20.0 software. Data were expressed as mean ± SEM. Differences between groups were considered significant at *P* < 0.05 by either *T* test or one-way ANOVA.

### Ethics approval and consent to participate

All animal and human experiments were approved by the Experimental Animal Ethics Committee of The Fourth Hospital of Hebei Medical University.

### Availability of data and materials

The data used to support the findings of this study are available from the corresponding author upon request.

## Supplementary Materials

Supplementary Figures
