# Supplementary material for: MicroRNA-181a-5p prevents the progression of esophageal squamous cell carcinoma in vivo and in vitro via the MEK1-mediated ERK-MMP signaling pathway
Source: Aging (Albany NY). 2022 Apr 25;14(8):3540–53. doi: 10.18632/aging.204028 (PMC9085224; doi:10.18632/aging.204028)
Supplement: Supplementary Figures [file aging-14-204028-s001.pdf]

SUPPLEMENTARY FIGURES

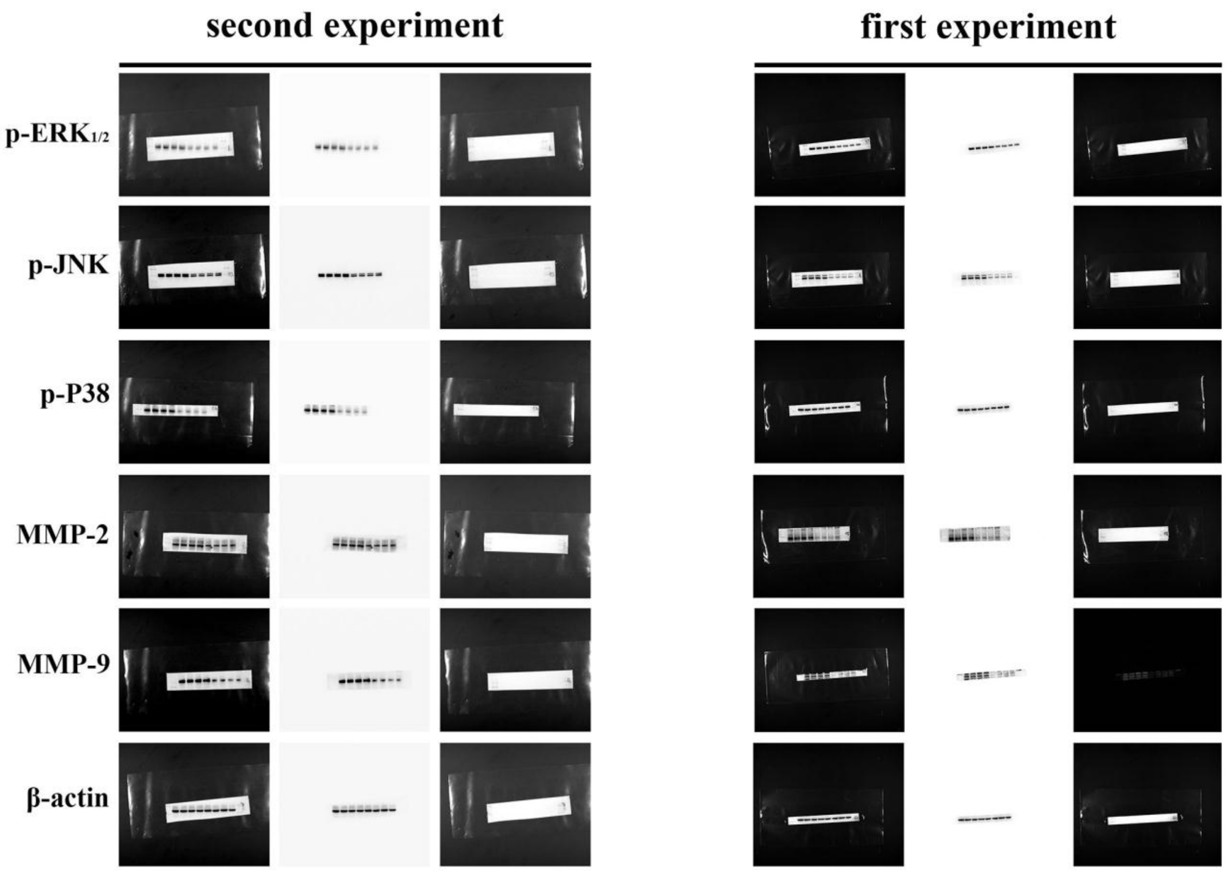

Supplementary Figure 1. Original western blot images for Figure 5.

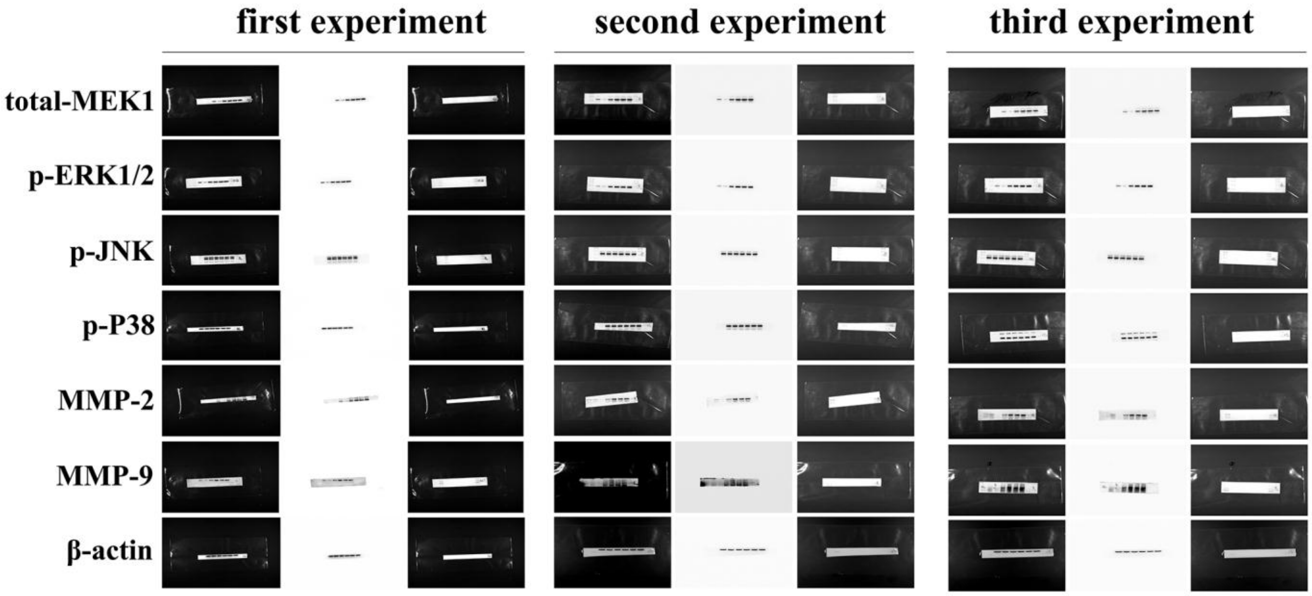

Supplementary Figure 2. Original western blot images for Figure 6.
